# No Reduction in Fare

**Published:** 1886-04

**Authors:** 


### NO REDUCTION IN FARE TO DELEGATES.

WE are in receipt of a note from Dr. J. H. Smith, Chairman of the Committee on Transportation, at Dallas, requesting us to announce that, "Gov. Brown, as spokesman of the Traffic Association, (in reply to a request for special rates to delegates to the State Medical Convention, to be held at Dallas, April 27, *et seq.*) informed the committee that no reduction will be given to delegates or others attending the Convention."

"The sins of the father shall be visited upon the children even unto the third generation." Even so it seems that the sins of a brother who sued the Santa Fe road for (imaginary) damages incurred while traveling on a complimentary pass, are to be visited upon the entire medical profession forever. Reduction was refused at Houston. The request was made while the above occurrence was fresh in the minds of the railroad officials, and it was supposed to be in resentment of the suit. We can imagine no other cause for refusing to physicians what is very generally extended to every other class of persons who meet in convention in Texas, and which, in other States, is always courteously given to them. This *may be* pluck, but it strikes us as bad judgment. Railroad companies have not too many friends at best, and will soon be regarded as public enemies—Ishmaels, whose hand is against all men.

